# Restoring the Clinical Learning Environment at Teaching Hospitals in Post-Assad Syria: A PHEEM Analysis and Recommendations

**DOI:** 10.12688/mep.20955.1

**Published:** 2025-04-14

**Authors:** Ghaith Alfakhry, Rama Kodmani, Munir Ghandour, Amer Al Munajjed, Rawan Khwanda

**Affiliations:** 1 Education Quality and Scientific Research Office, Al-Sham Private University, Damascus, Damascus Governorate, NA, Syria; 2 University Hospital of Dermatology and Venereology, Damascus University, Damascus, Damascus Governorate, NA, Syria; 3 Department of Internal Medicine, Damascus University, Damascus, Damascus Governorate, NA, Syria; 4 Department of Maxillofacial Surgery, Damascus Hospital, Damascus, Damascus Governorate, NA, Syria; 5 Department of Pediatrics, Damascus Hospital, Damascus, Damascus Governorate, NA, Syria

**Keywords:** Clinical Learning Environment, Postgraduate Hospital Education Environment Measure, PHEEM, Arabic PHEEM, post-Assad Syria.

## Abstract

**Introduction:**

After 54 years of Assad’s family dictatorship, Syria was finally free but, nonetheless, was in ruins. The health profession education training system is no exception, and there have been many indicators of this in the literature, but none of them have provided a systematic evaluation of the clinical learning environment (CLE) using validated approaches. Therefore, this study aimed to evaluate CLE at teaching hospitals in Damascus, Syria.

**Methods:**

This cross-sectional study was conducted in Damascus, Syria, during mid-2023, before Assad’s regime collapsed. The study population included all resident doctors working and training at any teaching hospital in Damascus. We used the recently validated 36-item PHEEM inventory as a data collection tool and added 10 extra items related to Syria’s specific context. There was also one open-ended question. We recruited participants from 14 different teaching hospitals using nonprobability sampling techniques.

**Results:**

A total of 1490 residents from 31 medical specialties participated in the study, which was approximately 37% of the total population at that time. The female participants comprised 50.7% (n=754) of the total sample. The Cronbach’s alpha was 0.925. The total PHEEM mean score was 72.4±21.4 (Max. 144). All the PHEEM domains showed significant shortcomings, except for learner engagement and social participation. The worst-scoring domains were external regulation, work culture, and living conditions, with scores of 48.5%, 40.8%, and 31.2%, respectively. The results of the additional 10 war-related items were important. One item showed that only 14% of respondents did not have plans to migrate. The written comments re-echo some of the PHEEM findings in more context.

**Discussion:**

This study draws a roadmap for clinical educators, lawmakers, and new leaders to make targeted reforms and investments to restore the clinical learning environment. There are major issues that not only render training residents suboptimal but also compromise residents' and patients’ safety.

## Introduction

After 54 years of dictatorship, Syria was finally free from Assad’s family rule, which left the country in ruins. The Syrian healthcare crisis is one of the major catastrophes internationally, with the World Health Organization (WHO) spokesperson saying that “ *Millions of Syrians lack access to basic medical care, and the risks in southern Syria are particularly urgent*’ ( Levant24, 2024). There is a major shortage of doctors, nurses, and other healthcare professionals. The healthcare infrastructure is in terrible condition with ambulances, medical equipment, and other essential medications in a very short supply ( Levant24, 2024). This places more emphasis on the efforts of the resident doctors. Nonetheless, there must be major reform in the personal, social and organizational structure of the clinical learning environment (CLE) which reality were mostly dictated by corrupt regime-affiliated doctors, staff and policymakers ( Alfakhry *et al.*, 2023a).

LE was defined by Josiah Macy Jr. Foundation consensus conferences include social interactions, organizational culture and structures, and physical and virtual spaces that surround and shape the learner’s experiences, perceptions, and learning” ( Gruppen *et al.*, 2018). Many questionnaires have been proposed to psychometrically measure CLE, one of which is the Postgraduate Hospital Educational Environment Measure (PHEEM) ( Roff *et al.*, 2005). This questionnaire has been widely used across the globe and has been translated into numerous languages, including Spanish, Danish, Greek, Persian, and Japanese, and most recently translated into Arabic ( Alfakhry *et al.*, 2023b; Aspegren *et al.*, 2007; Riquelme *et al.*, 2009; Shokoohi *et al.*, 2014; Taguchi *et al.*, 2008). The Arabic PHEEM has been psychometrically tested in Syria and consists of 36 items with a 5-factor structure ( Alfakhry *et al.*, 2024b).

There have been many indicators of deteriorating CLE in teaching hospitals in Syria. Some of these are the considerably high dropout rate, high burnout levels, and substantial daily workload in a resource- and staff-limited setting ( Alfakhry & Kodmani, 2024; Alhaffar *et al.*, 2019). Nonetheless, to the best of our knowledge, there has been no systematic evaluation of CLE. It is of utmost importance to critically evaluate teaching hospital environments during the Al-Assad period to direct new education policymakers and decision makers accurately to the right path in post-Assad Syria. Therefore, this study conducted a large-scale evaluation of the clinical learning environment in teaching hospitals in Syria’s capital, Damascus.

## Methods

### Study design

This was a cross-sectional study using a questionnaire survey method conducted in mid-2023. Ethical approval was granted by the Ethical Committee of Al-Sham Private University (no. 51655). Informed consent was obtained and participation was anonymous.

### Sample and settings

The population of this study was defined as all resident doctors from all specialties and stages working at any teaching hospital in Damascus. Specialty training programs in Syria are divided between the Ministry of Higher Education and the Ministry of Health; each has different affiliated hospitals. Generally, specialty training programs of the Ministry of Higher Education are better and of higher quality and are more competitive. Specialty training is four to five years for most specialties, with the exception of a few that have six or even seven years (such as cardiothoracic surgery). After completing their training, all trainees had to undergo an Imtiaz year, which is a mandatory service year that is now cancelled in late 2024 by the new administration. This study targeted all teaching hospitals in Damascus in order to recruit a good sample from all specialties. The following were the teaching hospitals targeted: 1) Ministry of Higher Education hospitals: Obstetrics University Hospital, Dermatology University Hospital, Children’s University Hospital, Al-Mousat University Hospital, National University Hospital (previously Al-Assad Hospital), Al Beiruni University Hospital, and Cardiothoracic Surgery University Hospital. 2) Ministry of Health hospitals: Eye Surgery Hospital, Ibn Alnafees Hospital, Red Crescent Hospital, Damascus Hospital, Al-Zahrawi Hospital, Renal Surgery Hospital, and Damascus Hospital for Heart Diseases and Surgery.

Most participants were recruited using snowball and convenience sampling, while about a quarter were recruited using a river sampling technique in which Google Forms was used to collect responses, targeting official social media groups on Facebook, Telegram, and WhatsApp dedicated to medical residents in Damascus ( Lehdonvirta *et al.*, 2021).

### Data collection

The data collection phase began in mid-June and ended on August 20, 2023. The validated Arabic version of PHEEM by Alfakhry *et al.* was used and is 50-item questionnaire ( Alfakhry *et al.*, 2024a; Alfakhry *et al.*, 2024b). The original 50-item Arabic PHEEM can be found on Figshare ( Alfakhry *et al.*, 2023c), whereas the English version will be displayed in Table 5 later. The last ten items are considered additional Syrian-context-specific items excluded in calculating the final PHEEM score; nonetheless, individual item results will be shown and discussed in this study. After psychometric analysis, only 36 items loaded significantly on the five factors ( Alfakhry *et al.*, 2024b). Items 14, 18, 37, and 38 (see ( Roff *et al.*, 2005)) were excluded due to insignificant factor loadings and were thus excluded from the analysis in this study ( Alfakhry *et al.*, 2024b). The PHEEM questionnaire used 5-point Likert scale (strongly disagree, disagree, unsure, agree, strongly agree) coded from 0 to 4. The total PHEEM score is 144 (36 items × 4 points), and there are five subscales that are original to the Arabic PHEEM: (perception of teachers, learners’ engagement and social participation, external regulation, work culture, and living conditions) ( Alfakhry *et al.*, 2024b). Table 1 provides a guide for interpreting the Arabic PHEEM and its subscales. Demographic data, including age, sex, specialization, hospital, and year of study, were also collected. There was also one open-ended question at the end of the PHEEM questionnaire: *Do you have anything to add about the clinical working environment at the hospital that you work in?* answers to this question were qualitative.

**Table 1.** A guide for interpretating the 36-item PHEEM scale and its subscales.

	Score	Interpretation
Total PHEEM	0–36	Very poor
	37–72	Plenty of problems
	73–108	More positive than negative but there’s room for improvement
	109–144	Excellent
Subscales		
Perception of teachers (10 items)	0–10	Very poor
	11–20	In need of some retraining
	21–30	Moving in the right direction
	31–40	Model teachers
Learner’s engagement and social participation (13 items)	0–13	Non-existent
	14–26	Not a pleasant place
	27–39	More pros than cons
	40–52	An engaging and active social environment
External regulations (5 items)	0–5	Disorganized place
	6–10	Many negative aspects
	11–15	Feeling fairly guided
	16–20	Organized and structured place
Work culture (6 items)	0–6	Physically and emotionally draining culture
	7–12	There are many issues that need to be addressed
	13–18	A more positive culture
	19–24	A positive work culture overall
Living conditions (2 items)	0–2	Miserable
	3–4	Not a nice place
	5–6	Not very bad
	7–8	Very good

### Data analysis

After reverse-coding the negatively phrased items, data normality checks were conducted based on skewness and kurtosis according to Hair, which has a rule of thumb of skewness =±2 and kurtosis =±7 to consider the variable normal ( Hair Jr *et al.*, 2010). The data were confirmed to be within an acceptable threshold; therefore, parametric tests were used. Google Forms was used to administer the electronic survey, Microsoft Excel 2019 was used to process the data, and IBM SPSS Statistics for Windows version 26 (IBM Corp., Armonk, N.Y., USA) was used to perform statistical analysis. Quantitative and qualitative data collected during the current study are shown in the figure Alfakhry *et al.* (2025).

## Results

In total, 1490 residents completed the PHEEM questionnaire. The majority of residents (1093) participated in the paper-based survey, while 397 (26.5%) completed the online-based survey. The characteristics of the sample are presented in Table 2. Approximately 37% (1490/3942) of all resident doctors in Damascus participated in the study; however, this was based on attendance records and resident-provided information. We were not able to obtain official records of residents from the hospitals during Al Assad’s period because of obstructive bureaucratic processes. Cronbach’s alpha for the 36-item PHEEM was 0.925 for 1349 valid cases (141 cases were excluded due to missing values).

**Table 2.** Characteristics of the study sample according to sex, age, training year and affiliation.

Sex	Male	Female				
No. (%)	735 (49.3%)	754 (50.7%)				
**Age ^1^**	**23–24**	**25–26**	**27–28**	**29–30**	**31–35**	
No (%)	135 (9%)	671 (45%)	454 (30%)	189 (13%)	32 (2%)	
**Training year ^2^**	**First**	**Second**	**Third**	**Fourth**	**Fifth**	**Imtiaz**
**N. (%)**	605 (40.6%)	301 (20.2%)	248 (16.6%)	175 (11.7%)	80 (5.3%)	73 (5%)
**Affiliation ^3^**	**Ministry of Higher ** **Education**	**Ministry of ** **Health**				
	841 (56.4%)	648 (43.5%)				

1 There are 4 participants older than 35 and there are 5 missing values, 2 There are 6 participants in the 6th year and 2 in the seventh. 3 There is one missing value.

There was no significant difference between male and female residents in the total PHEEM scale score ( *P*=0.337), and the effect size, as measured by Cohen’s d, was small ( *d*=0.05). Age was not correlated with the total PHEEM score according to Pearson’s correlation coefficient (r =-0.025, *P* = 0.169).

The PHEEM total score was 72.4±21.4 (N=1490, max. score: 144), and each subscale score is shown in Table 3. It can be noticed that all subscales are below the positive threshold except for ‘learner’s engagement and social participation’ domain. Table 4 shows the total PHEEM scores according to the participants’ medical specialty. Some specialties, such as pediatric surgery, infectious diseases, and cardiothoracic surgery, have very few residents; therefore, the number of participants is low. Generally, surgical specialties have lower PHEEM scores, with a few exceptions.

**Table 3.** Descriptive statistics of PHEEM scale and subscales (n=1490).

Subscales	Mean±SD	Score as (%)	Interpretation
Perception of teachers (max. 40)	20.3±7.8	50.7%	In need of some retraining
Learner’s engagement and social participation (max. 52)	30±8.1	57.6%	More pros than cons
External regulation (max. 20)	9.7±4	48.5%	Many negative aspects
Work culture (max. 24)	9.8±4.8	40.8%	There are many issues that need to be addressed
Living conditions (max. 8)	2.5±2.0	31.2%	Miserable
Total PHEEM score (max. 144)	72.4±21.4	50.2%	Plenty of problems

Underlined cells indicate a value of negative interpretation.

**Table 4.** PHEEM total score based on respondents’ medical specialty in a descending order (Underlined cells indicate a value of negative interpretation).

	Medical Specialty	N. of responses	PHEEM score (max. 144)
1	Pediatric surgery	4	102.3±6
2	Internal medicine: infectious diseases	6	87.3±17
3	Radiology	105	85.0±17
4	Psychiatry	11	84.1±16
5	Anaesthesia	20	81.0±30
6	Neurosurgery	25	80.5±21
7	Urological surgery	37	78.6±18
8	Obstetrics & Gynaecology	80	78.1±22
9	Laboratory Medicine	13	76.7±18
10	Dermatology	83	75.8±20
11	Ear, Nose and Throat	49	75.7±23
12	Oral maxillofacial surgery	28	74.9±25
13	Ophthalmology	120	74.4±22
14	Internal Medicine: Endocrine	50	73.7±14
15	Thoracic surgery	14	73.4±20
16	Internal Medicine: Gastroenterology	47	72.8±17
17	Oncology	29	72.3±15
18	Vascular surgery	10	70.8±37
19	Internal medicine: Nephrology	33	70.8±21
20	Internal medicine: General Medicine	173	70.8±19
21	Paediatrics	189	69.9±20
22	Internal medicine: Rheumatology	37	69.4±17
23	Orthopaedic surgery	37	67.2±20
24	Internal medicine: Neurology	46	66.0±20
25	Internal medicine: Haematology	29	65.9±12
26	Internal medicine: Cardiology	61	65.0±22
27	Internal medicine: pulmonology	56	64.3±24
28	General surgery	46	61.6±20
29	Emergency medicine	9	59.6±12
30	Cosmetic surgery	33	59.5±27
31	Cardiothoracic surgery	7	59.4±21

Individual item responses in their respective domains are shown in Table 5. It can be noticed that among the original PHEEM domains, perceptions of work culture and living conditions scored generally lower. Responses to item 42 “I plan to immigrate to another country” were as follows: 928 (63%) out of 1477 total response had plans to travel, 23% were undecided, and only 14% wanted to stay.

**Table 5.** Individual item mean scores of the PHEEM inventory organized according to the subscale. A five-point Likert scale is used (0=strongly disagree to 4=strongly agree) and items with negative phrasing were reverse coded so that the higher the score the more positive the response is. The word specialists refers to clinical teachers.

No.	Perception of teachers	N.	Mean±SD
35	Specialists have good mentoring skills	1474	2.2±1
40	Specialists promote an atmosphere of mutual respect	1477	2.3±1
28	Specialists have good teaching skills	1478	2.2±1
10	Specialists have good communication skills	1473	2.2±1
23	Specialists perform their tasks in an organized manner	1476	2.1±1
31	Specialists are accessible	1478	2.1±1
15	Specialists are enthusiastic	1479	1.9±1.1
19	Specialists provide advice regarding my professional career	1473	1.7±1.1
39	The clinical teachers provide me with good feedback on my strengths and weaknesses.	1472	1.6±1.3
21	The curriculum is appropriate to meet my educational needs	1478	1.6±1
	Perception of Learner’s engagement and social participation		
16	I have good collaboration with other doctors in my grade	1478	3.0±0.8
7	There is discrimination based on (Ethnicity, religion, socioeconomic status, age) in this post*	1477	2.6±1.1
29	I feel part of a team working here	1479	2.6±1
22	I get regular feedback from seniors	1473	2.5±1.3
30	I have opportunities to acquire the appropriate practical procedures for my grade	1475	2.5±1
5	Level of responsibilities is appropriate to my year of study	1489	2.4±1.1
13	There is sex discrimination in this post*	1479	2.3±1.4
34	The training in this post makes me feel ready to be a SpR/Consultant	1475	2.2±1
12	I am able to participate actively in educational events	1474	2.2±1
33	Senior doctors make use of opportunities to teach me	1468	2.1±1
27	I have enough clinical learning opportunities for my needs	1472	2.0±1
6	I have good clinical supervision at all times	1478	2.0±1.1
36	I get a lot of enjoyment out of my present job	1476	2.0±1.1
	Perception of external regulation		
2	Specialists set the job responsibilities required of me	1486	2.52±1
9	There is an informative Junior Doctors handbook	1484	2.14±1.2
4	I had an informative induction programme	1488	2.11±1.2
3	Time dedicated for the educational process is of utmost importance in the hospital	1487	1.93±1.2
1	I have a contract of employment that provides information about hours of work	1468	1.09±1
	Perception of work culture		
24	I feel physically safe within the hospital environment	1476	1.95±1.1
17	Number of working hours at the hospital is acceptable	1482	1.78±1.3
32	My workload in this job is fine	1478	1.75±1.2
11	I am being summoned for unnecessary matters when I am on call*	1476	1.60±1.1
8	I have to perform inappropriate tasks*	1478	1.51±1.2
25	There is a no-blame culture in this post when some error occurs	1479	1.31±1.1
	Perception of living conditions		
26	There are adequate catering facilities when I am on call	1474	1.35±1.1
20	This hospital has good quality accommodation for junior doctors, especially when on call	1479	1.16±1.2
	Additional items related to medical education in war settings		
41	The hospital provides adequate medical equipment and supplies	1473	1.2±1
48	Patients are aggressive towards doctors*	1476	1.9±1
45	I feel constantly stressed in this hospital*	1475	1.7±1.1
50	There are adequate educational references and resources for my need in this hospital	1478	1.6±1.1
46	My physical health was affected negatively in this job*	1476	1.5±1.1
47	There is shortage in the medical staff in this hospital*	1474	1.4±1.2
44	I would like to work in this hospital after finishing my training	1474	1.2±1
42	I plan to immigrate to another country*	1477	1.2±1.1
43	The hospital pharmacy contains necessary medications and medical materials*	1472	1.0±0.9
49	The educational aspect of residency is overlooked due to the healthcare workload	1477	0.9±0.9

### Residents’ voice

Participants were asked to comment on anything they would like to add to the clinical learning environment. There were 177 comments, most of which fell under the three main themes of workload, curriculum, and living conditions.

There were 32 comments related to workload, discussing how working overtakes learning at the hospital, and “no time to study” said one resident. Some of the descriptions of the workload were: “inhumane,” “very draining,” and “physically intense and unbearable.” One participant commented,

*Workload doesn’t just affect doctors, it also affects patients. Sleep-depraved doctors could harm the patient by mistake and the nurses are the worst part of the hospital working staff.*

It was mentioned that residents usually do nurses’ work, who usually have more privilege than residents and are generally not cooperative in doing their own tasks. One participant said, ‘ *Role of nursing staff is terrible, the doctor does his job and the nursing staff job.”* Their salary was mentioned multiple times, and at the time of data collection, it was very dreadful. A good proportion of responses were about the curriculum and teaching, which highlighted many issues, such as shortage of teaching staff, over-recruitment of residents, substandard training, negligence of teaching duties, disorganized learning environment, lack of standard clinical protocols, and lack of mutual respect between residents.

Living conditions was a topic that many participants commented on highlighting the very dreadful status of residents’ dorms which have substandard hygiene, lacks conditioning, and does not have enough beds. Restrooms have also been criticized for hygiene. The food offered by the hospital was described as inappropriate for human consumption.

Other important themes were the shortage of medical equipment and materials, and psychological and physical well-being. There is a serious shortage of essential medical equipment, and many existing devices are outdated, unreliable, and malfunctioning. According to the participants’ comments, some emergency and essential medical materials and drugs were unavailable at hospitals. One response echoed by others.

*There is a serious shortage of medical equipment. Most drugs even emergency ones are unavailable at the hospital. Some equipment are very old and does not work very well (like ECG and Echo equipment).*

Many residents described that their psychological health was affected by the toxic work environment, underappreciation, lack of mutual respect, intense workload, and long working hours. These conditions caused many to experience burnout, regret, mental instability, stress, or even led them to leave the residency programme together. Physical well-being is also affected by some patients’ physical and verbal aggression.

## Discussion

This study aimed to provide a critical systematic evaluation of the clinical learning environment at teaching hospitals in Syria’s capital, Damascus, in order to provide a clear pathway for change in post-Assad Syria. Using the validated Arabic version of PHEEM with the addition of ten extra items to fit Syria’s war context ( Alfakhry *et al.*, 2024b; Alfakhry *et al.*, 2024c). This study showed that many critical issues need to be addressed in almost all aspects of the clinical learning environment, especially with regard to work culture and living conditions. The ten extra war-related items showed that participants perceived a major shortage in medical staff, equipment, and supplies, in addition to the lack of educational resources. In addition, participants’ physical and mental health appeared to be considerably affected by their jobs. Qualitative findings corroborated quantitative findings showing a draining workload, substandard training and disorganized curriculum, low-quality unhygienic living conditions, shortage of medical equipment and supplies, and risks to physical and mental health.

A comparison with other PHEEM studies conducted worldwide shows some interesting similarities and differences. Many studies, including our study, have shown that low-quality catering facilities and accommodation ( Aalam *et al.*, 2018; Bari *et al.*, 2018), excessive workload ( Fisseha *et al.*, 2021; Flaherty *et al.*, 2016), and lack of protected study time ( Vieira *et al.*, 2016) are common issues in the clinical learning environment in many contexts, whether in first-world countries or developing countries. However, there are some issues that are unique to our study, such as the unsafety of the hospital environment, having to perform inappropriate tasks, sexual discrimination, and other kinds of discrimination. Furthermore, the significant lack of medical equipment, supplies, materials, and staff in the hospital is an unusual finding, as educational environment studies are not usually reported. Only one study in Ethiopia has reported a shortage of medical equipment ( Fisseha *et al.*, 2021).

In war and conflict settings, medical care services and professional health training systems become largely disrupted due to a multitude of factors. A recent scoping review of maintaining medical education in war settings found five main barrier categories: curriculum, personnel, wellness, resources, and oversight, most of which can be observed in our study ( Dobiesz *et al.*, 2022). Whether it lacks a clear curriculum, decreased quality of training, low number of supervisors instructors, safety issues, physical and psychological toll on residents, overstrained hospital system, lack of medical supplies and equipment, increase in resident numbers, and attrition of faculty and students who may be drafted into the military, flee due to safety or financial concerns, and look for better career opportunities abroad ( Dobiesz *et al.*, 2022). All of the above apply to the Syrian war-ravaged context.

This study has important implications for researchers, clinical educators, and decision-makers. First, this study showed the utility, applicability, and suitability of Arabic PHEEM in a unique Syrian context. Researchers can use this invaluable validated tool to evaluate the efficacy of any interventions or reforms targeting the clinical learning environment in Post-Assad Syria. Second, clinical educators need to consider the areas of improvement highlighted in this study to develop targeted educational interventions in accordance with available resources. Third, decision makers have to revise educational and professional policies in order to address some fundamental issues that need addressing, such as the job contract, determining residents’ job responsibilities, revising hours of work, protecting educational time, and other regulatory aspects of the residency system.

Post-Assad Syria is witnessing many fast-track changes in the healthcare system, including changes in the leadership of teaching hospitals, but actual changes are yet to be implemented. Many non-governmental organizations are now helping provide hospitals with essential medical equipment and supplies. Systematic changes in how teaching hospitals are run are yet to occur and need to be planned in accordance with available evidence and quality evaluations on multiple levels, including infrastructure, patient care, human resources, resident training, faculty retraining, capacity building, curriculum reform, etc.

### Strengths and limitations

This study is by far and to the best of our knowledge the second biggest PHEEM study in terms of sample size in the world and the first study applying the validated Arabic PHEEM. This is the first systematic large-scale evaluation of the clinical learning environment in Syria. The sample size was approximately 37% of the total population, including residents from all specialities in teaching hospitals affiliated with the Ministry of Health and the Ministry of Higher Education in Damascus. This study also provided a qualitative account of residents lived experiences. This study is of utmost importance to the new green Syria, as it paints a roadmap of change in the postgraduate education system. The inability to use random sampling methods somewhat limits the generalizability of our findings; however, the large sample size compensates for this limitation.

## Conclusion

The clinical learning environment at teaching hospitals in Syria has been exhausted since over a decade of war. There are major issues that not only render training residents suboptimal but also compromise residents’ and patients’ safety. To rebuild the Syrian healthcare postgraduate training system, major investments must be made in hospital infrastructure, faculty and staff recruitment training, and curriculum reform. Funds need to be directed wisely to the most needed areas first, and reforms need to be planned in accordance with available evidence. Future research needs to continue to evaluate the impact of current and future interventions and reforms taking place in post-Assad Syria on the clinical learning environment.

## Ethics and consent

This study was approved by the Ethics Committee of Al-Sham Private University (no. 51655) on February 11 ^th^, 2023. Verbal informed consent was obtained from all participants; the ethics committee approved verbal informed consent for practicality and because the study is considered low-risk. verbal consent was obtained from all participants without audio or written recording. This approach was chosen due to cultural sensitivities in Syria, where individuals may be hesitant to provide written consent out of concern for potential legal or political implications. The fear of signing official documents, particularly in sensitive contexts, is a common barrier to participation in research. To ensure ethical integrity, participants were thoroughly informed about the study's purpose, their voluntary participation, and their right to withdraw at any time. The research team documented the consent process in field notes, ensuring that all ethical considerations were met while respecting the participants’ comfort and security.All methods were performed in accordance with Helsinki declaration.

## Consent for publication

Not applicable.

## Data Availability

Figshare: Restoring the clinical learning environment at teaching hospitals in post-Assad Syria: A PHEEM analysis and recommendations. https://doi.org/10.6084/m9.figshare.28418339.v2 ( Alfakhry *et al.*, 2025) The project contains the following underlying data: PHEEM Syria.sav Qualitative data.xlsx Data are available under the terms of the Creative Commons Attribution 4.0 International license (CC-BY 4.0).
