# Supplementary material for: Restoring the Clinical Learning Environment at Teaching Hospitals in Post-Assad Syria: A PHEEM Analysis and Recommendations
Source: MedEdPublish (2016). 2025 Aug 5;15:15. Originally published 2025 Apr 14. [Version 2] doi: 10.12688/mep.20955.2 (PMC12082068; doi:10.12688/mep.20955.2)
Supplement: Peer review history

# Reviewer report: Reviewer response for version 2

**DOI:** 10.21956/mep.22735.r42745

**Published:** 2025-08-19

**Authors:** Kathy Chappell

**Affiliations:** 1 Accreditation Commission for Education in Nursing, Georgia, USA

This is a fascinating yet sad reflection of the current clinical learning environment in post-Assad Syria and I commend the authors for their commitment to describing the current state and advocating for improvement.  The description of residents regarding nursing was particularly interesting to me. I believe that the results could be enhanced or perhaps a follow up study conducted that better describes the nursing perspective on post-graduate medical resident training and the clinical learning environment.  The discussion and recommendations might include the opportunity to collaborate across the professions, creating interprofessional work teams that better align existing resources with need.

Have any limitations of the research been acknowledged?

Yes

Is the work clearly and accurately presented and does it cite the current literature?

Yes

If applicable, is the statistical analysis and its interpretation appropriate?

I cannot comment. A qualified statistician is required.

Are all the source data underlying the results available to ensure full reproducibility?

Yes

Is the study design appropriate and is the work technically sound?

Yes

Are the conclusions drawn adequately supported by the results?

Partly

Are sufficient details of methods and analysis provided to allow replication by others?

Yes

Reviewer Expertise:

interprofessional education, nurse residency, team-based education, research, quality

I confirm that I have read this submission and believe that I have an appropriate level of expertise to confirm that it is of an acceptable scientific standard.

# Reviewer report: Reviewer response for version 2

**DOI:** 10.21956/mep.22735.r42727

**Published:** 2025-08-19

**Authors:** Patrícia Lofêgo Gonçalves

**Affiliations:** 1 Universidade Federal do Espírito Santo, Vitoria, State of Espírito Santo, Brazil

The authors clarified the meaning of the asterisk in the caption to Figure 5, making it easier to understand.

Have any limitations of the research been acknowledged?

Yes

Is the work clearly and accurately presented and does it cite the current literature?

Yes

If applicable, is the statistical analysis and its interpretation appropriate?

Yes

Are all the source data underlying the results available to ensure full reproducibility?

Yes

Is the study design appropriate and is the work technically sound?

Yes

Are the conclusions drawn adequately supported by the results?

Yes

Are sufficient details of methods and analysis provided to allow replication by others?

Yes

Reviewer Expertise:

gastroenterology, hepatology, medical education

I confirm that I have read this submission and believe that I have an appropriate level of expertise to confirm that it is of an acceptable scientific standard.

# Reviewer report: Reviewer response for version 1

**DOI:** 10.21956/mep.22442.r41528

**Published:** 2025-05-13

**Authors:** Ngozi Chukwu

**Affiliations:** 1 University of Abuja, Abuja, Nigeria

1. TITLE AND RELEVANCE: The study size is concise, relevant and appropriately reflect the subject matter which addresses a critical sector of the healthcare industry. It promotes insight in context of conflict or systemic challenges

 2. OBJECTIVES & PROBLEM STATEMENT: The research objectives were clearly defined and problem statement is well articulated. The authors incorporated relevant and up-to-date literature, ensuring the study is well grounded in current discourse

 3. METHODOLOGY: The methodology was systematically presented. The use of the Postgraduate Hospital Educational Environment Measure (PHEEM), a validated and widely recognized tool enhances the credibility of the study (Roff et al., 2005). The instrument was appropriately applied to assess the educational climate, lending reliability to the data collected.

 4. FINDINGS & RECOMMENDATION: Findings are logically organized and presented with clarity. The recommendations offered a constructive and contextually appropriate on a region impacted with conflict. Furthermore, the study calls for additional research in conflict affected areas is timely and warranted

 5. ACKNOWLEDGEMENT OF LIMITATION: The authors acknowledged the limitations in the study indicating an awareness of the constraints in scope. This transparency strengthens the overall scholarly integrity of the work

 6. ETHICAL CONSIDERATIONS: A significant ethical issue occurred in the treatment of the participant confidentiality. The study obtained verbal informed consent, the direct inclusion of the respondents response compromised participants anonymity. According to the declaration of Helsinki & guidelines by the Council for International Organizations of Medical Sciences (CIOMS), researchers must ensure that identifiable information is not disclosed without explicit consent (CIOMS, 2016; WMA, 2013) 

 RECOMMENDATION: The authors should consider paraphrasing responses or providing quotes where necessary to protect confidentiality. Future studies should implement anonymization protocols when using direct quotes.

 CONCLUSION: Generally, the study is a valuable contribution to the discipline, providing relevant findings and implications for medical education in conflict affected settings. However, attention to ethical rigor is essential to uphold the trust and safety of research participants      

Have any limitations of the research been acknowledged?

Yes

Is the work clearly and accurately presented and does it cite the current literature?

Yes

If applicable, is the statistical analysis and its interpretation appropriate?

I cannot comment. A qualified statistician is required.

Are all the source data underlying the results available to ensure full reproducibility?

Yes

Is the study design appropriate and is the work technically sound?

Yes

Are the conclusions drawn adequately supported by the results?

Yes

Are sufficient details of methods and analysis provided to allow replication by others?

Yes

Reviewer Expertise:

Nursing research in clinical practice and nursing education/ maternal and neonatal child care/ Reproductive health

I confirm that I have read this submission and believe that I have an appropriate level of expertise to confirm that it is of an acceptable scientific standard.

# Reviewer report: Reviewer response for version 1

**DOI:** 10.21956/mep.22442.r41522

**Published:** 2025-04-21

**Authors:** Patrícia Lofêgo Gonçalves

**Affiliations:** 1 Universidade Federal do Espírito Santo, Vitoria, State of Espírito Santo, Brazil

This is a cross-sectional study on the educational environment of medical residency programs in Syria through the application of the Arabic version of the PHHEM questionnaire plus 10 additional items related to the Syrian context and an open-ended question. The study demonstrated that the educational environment presents many problems, mainly related to external regulation, work culture and residents' living conditions.

 The study reflects the local reality of medical residency programs in Syria and highlights critical areas that need to be improved. The inclusion of an open-ended question was of great value allowing for a spontaneous report on situations experienced and how residents feel in teaching hospitals.

 The article is well structured and well written. It includes a large number of participants, although the sample was not randomized. The tables are clear and explanatory. However, in Table 5 some items are marked with an asterisk, but the meaning of this asterisk is not explained in the table caption.

Have any limitations of the research been acknowledged?

Yes

Is the work clearly and accurately presented and does it cite the current literature?

Yes

If applicable, is the statistical analysis and its interpretation appropriate?

Yes

Are all the source data underlying the results available to ensure full reproducibility?

Yes

Is the study design appropriate and is the work technically sound?

Yes

Are the conclusions drawn adequately supported by the results?

Yes

Are sufficient details of methods and analysis provided to allow replication by others?

Yes

Reviewer Expertise:

gastroenterology, hepatology, medical education

I confirm that I have read this submission and believe that I have an appropriate level of expertise to confirm that it is of an acceptable scientific standard.
